# Clinicopathological Study of Fibroepithelial Lesions of the Breast in a Tertiary Care Hospital in South India

**DOI:** 10.7759/cureus.64043

**Published:** 2024-07-07

**Authors:** Indhu Gunasekaran, Karthik Sigamani

**Affiliations:** 1 Pathology, Karpaga Vinayaga Institute of Medical Sciences and Research Centre, Chengalpattu, IND

**Keywords:** malignant, borderline, benign, tubular adenoma, complex fibroadenoma, cellular fibroadenoma, biphasic, phyllodes tumour, fibroadenoma, females

## Abstract

Introduction

Fibroepithelial lesions of the breast mainly include fibroadenoma and phyllodes tumors with overlapping morphological features and varied clinical behavior. This study aims to determine the histopathological spectrum of fibroepithelial lesions of the breast in a tertiary care hospital.

Methods

This is a cross-sectional study that was carried out in the pathology department from 1^st^ January 2015 to 31^st^ December 2023. Relevant data of all fibroepithelial lesions reported during the study period were retrieved from the medical records, tabulated, and analyzed. The Pearson chi-square test was used to determine the significant association between the various clinicopathological parameters of fibroepithelial lesions. A p-value of less than 0.05 was taken as statistically significant.

Results

Out of a total of 195 fibroepithelial lesions, 185 (95%) were fibroadenoma, 07 (3.5%) were phyllodes tumors, and three (1.5%) were fibroadenoma with tubular adenoma. The most common age group was 21 to 40 years, with the majority of phyllodes tumors being more than 5 cm in size compared to fibroadenomas. The association between the clinicopathological characteristics such as age of patients, tumor size, and histological grade was statistically insignificant in this study.

Conclusions

The implementation and usage of morphological diagnostic criteria will help in diagnosing and categorizing this broad group of fibroepithelial lesions, thereby facilitating appropriate treatment for patients.

## Introduction

Fibroepithelial lesions of the breast include a spectrum of lesions arising from the terminal duct lobular unit (TDLU) containing duct epithelial elements along with stromal components. They result from the neoplastic proliferation of the specialized intralobular breast stroma, resulting in distortion and incorporation of the ducts within the tumor mass [1]. Fibroepithelial lesions of the breast include two major categories, namely fibroadenoma and phyllodes tumor, that differ in morphological features and clinical behavior [1]. Fibroadenoma is the most common benign fibroepithelial tumor of the breast, while phyllodes tumor accounts for only 0.3 to 0.9% of the primary neoplasms of the breast [1].

Fibroadenomas are estrogen-sensitive benign fibroepithelial tumors occurring in females during the reproductive age group, with a peak incidence between 20 and 35 years of age [2]. Fibroadenomas usually present as a slow-growing, painless, firm, and freely mobile lump in the breast, which can be single or multiple, ranging in size from 3 cm to 10 cm. Fibroadenomas include multiple histological variants like myxoid fibroadenoma, complex fibroadenoma, cellular fibroadenoma, and juvenile fibroadenoma, with 0.1% of cases showing malignant transformation, especially in the complex fibroadenoma variant [2].

Phyllodes tumor was initially described as cystosarcoma phyllodes tumor owing to its leaf-like morphological pattern, frequent cystic change, and fleshy cut surface [3]. These tumors occur in women in the age group of 30 to 70 years, with a mean age of 44 years [4]. These tumors clinically present as a rapidly growing, firm to hard, large breast lump usually more than 4 cm in size that may ulcerate into the skin or extend into the chest wall [4]. Phyllodes tumors are characterized by overgrowth of the stromal component and are subclassified into three histological grades, namely benign, borderline, and malignant, based on the histological characteristics of the tumor [2]. Malignant phyllodes tumor shows an aggressive clinical course with a risk of distant metastasis [4]. The broad group of fibroepithelial lesions of the breast poses serious diagnostic challenges, especially in core needle biopsy samples, owing to overlapping histological features in the limited tissue examined [5]. Hence this study aims to determine the histopathological spectrum of fibroepithelial lesions of the breast in a tertiary care hospital.

## Materials and methods

This is a cross-sectional study carried out in the pathology department of Karpaga Vinayaga Institute of Medical Sciences and Research Centre, Chengalpattu district, TN, IND. The study period was nine years, i.e., from 1st January 2015 to 31st December 2023. All cases of fibroepithelial lesions of the breast reported in histopathology during the study period were included in the study. A purposive sampling technique was used for the selection of desired samples according to the inclusion criteria, and the sample size collected was 100 [1]. The study was approved by the Institutional Ethics Committee for Human Studies, Karpaga Vinayaga Institute of Medical Sciences and Research Centre (approval no.: KIMS/PG/06/09/2023).

Relevant clinical and pathological data of all fibroepithelial lesions reported during the study period were retrieved from the medical records of the Department of Pathology. Hematoxylin and eosin (H&E)-stained slides of the paraffin tissue blocks made from the formalin-fixed specimens of all the fibroepithelial lesions were retrieved and reviewed. Phyllodes tumors were graded into benign, borderline, and malignant as per the WHO criteria that include the degree of stromal cellularity, stromal overgrowth, cellular atypia, increased mitotic activity, and nature of the tumor margins [1].

All the findings were entered in Microsoft Excel (Microsoft® Corp., Redmond, WA, USA) and analyzed using SPSS Statistics version 23.0 (IBM Corp., Armonk, NY, USA). Results were expressed as frequency and percentage. The Pearson chi-square test was used to determine the association between the various clinicopathological parameters such as patient’s age, tumor size, and histological grade of the fibroepithelial lesions. A p-value of less than 0.05 was taken as statistically significant.

## Results

A total of 195 cases of fibroepithelial lesions of the breast were encountered in this study, out of which 185 (95%) were fibroadenoma and its variants, seven (3.5%) were phyllodes tumors, and three (1.5%) were fibroadenoma with tubular adenoma. The variants of fibroadenoma encountered in this study were usual fibroadenoma 116 (59.5%), complex fibroadenoma 65 (33.4%), and cellular fibroadenoma 4 (2.1%). Phyllodes tumors included three (1.5%) benign, one (0.5%) borderline, and three (1.5%) malignant cases (Table 1).

**Table 1:** Histopathological distribution of fibroepithelial lesions of the breast

Fibroepithelial lesions		Frequency (n)	Percentage	Total: n (%)
Fibroadenoma and its variants	Usual fibroadenoma	116	59.5%	185 (95%)
	Complex fibroadenoma	65	33.4%	
	Cellular fibroadenoma	04	2.1%	
Phyllodes tumour	Benign	03	1.5%	07 (3.5%)
	Borderline	01	0.5%	
	Malignant	03	1.5%	
Fibroadenoma with tubular adenoma		03	1.5%	03 (1.5%)
Total		195	100%	195 (100%)

**Figure 1:**
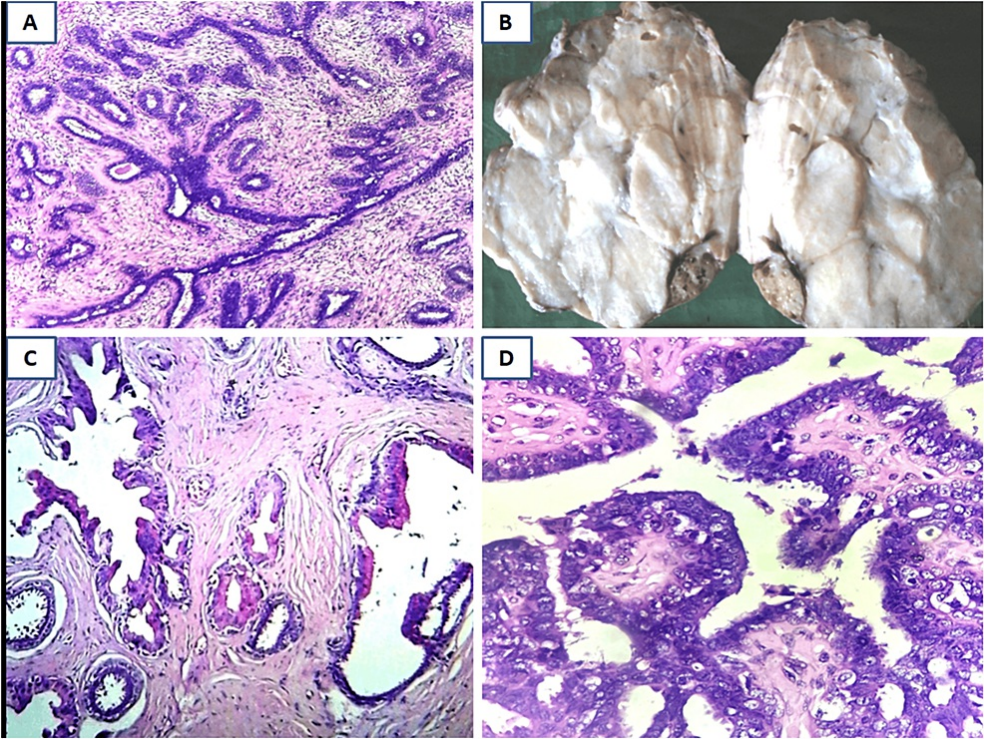
Histopathological features of fibroadenoma A: Usual fibroadenoma showing biphasic stromal and epithelial components (H&E, 10x); B: Gross, giant complex fibroadenoma with lobulated, gray-white cut surface; C: Complex fibroadenoma showing cysts, apocrine metaplasia, and sclerosing adenosis (H&E, 10x); D: Complex fibroadenoma showing papillary apocrine metaplasia (H&E, 40x) H&E: Hematoxylin and eosin

Fibroadenomas are characterized by a circumscribed mass with a lobulated gray-white cut surface showing slit-like spaces. On microscopic examination, fibroadenomas exhibit biphasic proliferation of glandular and stromal components in an intracanalicular or pericanalicular pattern. Complex fibroadenomas exhibit cysts larger than 3 cm in size, sclerosing adenosis, papillary apocrine metaplasia, or epithelial calcification (Figure 1).

**Figure 2:**
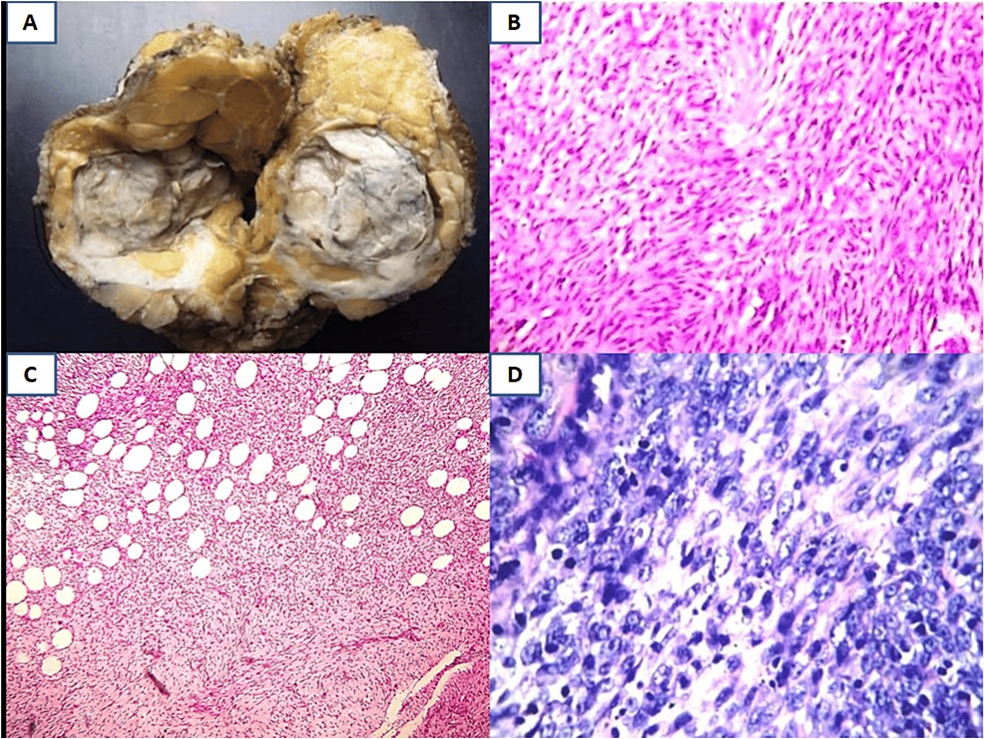
Histopathological features of a phyllodes tumor A: Gross, benign phyllodes tumor with fleshy, cut-cabbage appearance; B: Benign phyllodes tumor showing diffuse stromal hypercellularity (H&E, 40x); C: Malignant phyllodes tumor showing invasive margins (H&E, 10x); D: Malignant phyllodes tumor showing pleomorphic nuclei with brisk mitoses (H&E, 40x) H&E: Hematoxylin and eosin

Phyllodes tumors are fibroepithelial lesions with a leaf-like growth pattern. Grossly, these tumors are often larger with a characteristic cut-cabbage appearance. Benign phyllodes tumors are characterized by mild stromal hypercellularity without stromal overgrowth, mild nuclear pleomorphism, and mitoses less than 5/10 high power fields. Malignant phyllodes tumors show infiltrative margins, stromal overgrowth associated with stromal hypercellularity, marked nuclear pleomorphism, and brisk mitoses with more than 10/10 high power fields (Figure 2).

The age distribution of fibroepithelial lesions of the breast revealed that the majority of fibroadenomas (119, 63.3%) and phyllodes tumors (five, 71.4%) occurred between 21 and 40 years of age, with no cases encountered in females more than 60 years of age. Unlike fibroadenomas, none of the phyllodes tumors were encountered in less than 20 years of age (Table 2).

**Table 2:** Age distribution of fibroepithelial lesions

Fibroepithelial lesions		Age distribution			
		≤20 years (n)	21-40 years (n)	41-60 years (n)	>60 years (n)
Fibroadenoma and its variants (n=188)	Cellular fibroadenoma	3	1	0	0
	Complex fibroadenoma	17	42	6	0
	Usual fibroadenoma	34	75	7	0
	Fibroadenoma with tubular adenoma	2	1	0	0
	Total n (%)	56 (29.8%)	119 (63.3%)	13 (6.9%)	0 (0.0%)
Phyllodes tumor (n=7)	Benign phyllodes tumor	0	2	1	0
	Borderline phyllodes tumor	0	0	1	0
	Malignant phyllodes tumor	0	3	0	0
	Total n (%)	0 (0.0%)	5 (71.4%)	2 (28.6%)	0 (0.0%)

In the present study, the majority of fibroadenomas (105, 55.9%) were observed in the right breast while phyllodes tumors (six, 85.7%) were more common in the left breast (Table 3).

**Table 3:** Site distribution of fibroepithelial lesions

Fibroepithelial lesions		Site distribution		
		Bilateral breast (n)	Left breast (n)	Right breast (n)
Fibroadenoma and its variants (n=188)	Cellular fibroadenoma	0	2	2
	Complex fibroadenoma	5	25	35
	Usual fibroadenoma	6	44	66
	Fibroadenoma with tubular adenoma	0	1	2
	Total n (%)	11 (5.8%)	72 (38.3%)	105 (55.9%)
Phyllodes tumor (n=7)	Benign phyllodes tumor	0	3	0
	Borderline phyllodes tumor	0	0	1
	Malignant phyllodes tumor	0	3	0
	Total n (%)	0 (0.0%)	6 (85.7%)	1 (14.3%)

In the current study, the majority of fibroadenomas (171, 91%) were less than 5 cm in size, while four (57.1%) phyllodes tumors were more than 10 cm in size grossly (Table 4). Thus, patients with phyllodes tumors often presented with a larger breast lump compared to those with fibroadenomas.

**Table 4:** Size distribution of fibroepithelial lesions

Fibroepithelial lesions		Size distribution		
		<5cm (n)	5-10cm (n)	>10cm (n)
Fibroadenoma and its variants (n=188)	Cellular fibroadenoma	4	0	0
	Complex fibroadenoma	58	5	2
	Usual fibroadenoma	107	8	1
	Fibroadenoma with tubular adenoma	2	1	0
	Total n (%)	171 (91%)	14 (7.4%)	3 (1.6%)
Phyllodes tumor (n=7)	Benign phyllodes tumor	0	2	1
	Borderline phyllodes tumor	0	0	1
	Malignant phyllodes tumor	0	1	2
	Total n (%)	0 (0.0%)	3 (42.9%)	4 (57.1%)

The association between the age of the patients and tumor size showed statistically insignificant association in case of both fibroadenoma (p=0.210) and phyllodes tumor (p=0.809) in the present study, though tumors larger than 5 cm in size were often seen in patients less than 20 years of age in case of fibroadenoma compared to that of phyllodes tumors, which often presented as a larger tumor size in the group aged between 21 and 40 years (Tables 5-6).

**Table 5:** Association between the age of the patients and tumor size of fibroadenomas

Age (years)	Tumor size of fibroadenomas			Total n (%)	Pearson chi-square test
	<5cm (n)	5-10cm (n)	>10cm (n)		
≤20	47	08	01	56 (29.8%)	Chi-square value: 5.864 (p-alue=0.210)
21-40	112	05	02	119 (63.3%)	
41-60	12	01	0	13 (6.9%)	
Total	171	14	03	188 (100%)	

**Table 6:** Association between the age of the patients and the size of phyllodes tumors

Age (Years)	Size of phyllodes tumors			Total n (%)	Pearson chi-square test
	<5cm (n)	5-10cm (n)	>10cm (n)		
≤20	0	0	0	0 (0.0%)	Chi-square value: 0.058 (p-value=0.809)
21-40	0	2	3	5 (71.4%)	
41-60	0	1	1	2 (28.6%)	
Total	0	3	4	7 (100%)	

The common age group for phyllodes tumors was 21 and 40 years, which accounted for five (71.4%) cases, among which three (42.8%) were malignant and two (28.6%) were benign tumors. Thus, there was no significant association between histological grade and age group of patients in the case of phyllodes tumors (p=0.155) in this study. Out of seven cases of phyllodes tumors, three (42.9%) benign and three (42.9%) malignant grade tumors were more than 5 cm in size. Thus there was no significant correlation between tumor size and histological grade of phyllodes tumors (p=0.459) in the present study (Table 7).

**Table 7:** Association of histological grade with the age of the patients and size in phyllodes tumors

Age (years)	Histological grade of phyllodes tumours			Total n (%)	Pearson chi-square test
	Benign (n)	Borderline (n)	Malignant (n)		
≤20	0	0	0	0 (0.0%)	Chi-square value: 3.733 (p-value=0.155)
21-40	2	0	3	5 (71.4%)	
41-60	1	1	0	2 (28.6%)	
Tumor size (cm)					
<5	0	0	0	0 (0.0%)	Chi-square value: 1.556 (p-value=0.459)
5-10	2	0	1	3 (42.9%)	
>10	1	1	2	4 (57.1%)	

## Discussion

The two major fibroepithelial lesions of the breast are fibroadenoma and phyllodes tumors with overlapping histomorphological features and varied clinical behavior. These are biphasic tumors characterized by the proliferation of epithelial and stromal components [4]. Fibroadenoma and its variants are benign fibroepithelial tumors of the breast, while phyllodes tumors include benign, borderline, and malignant histological subtypes.

Fibroadenoma was the most common fibroepithelial epithelial tumor in the present study, accounting for 95% of the total cases, with the majority of cases (119, 63.3%) occurring in females of reproductive age group (21 to 40 years). These findings were in concordance with the studies of Yadav et al. [1], Al-Atrooshi [2], Wani et al. [6], and Patil et al. [7]. Fibroadenomas commonly involved the right breast in 55.9% of cases, with 91% of them presenting as a small, mobile, and non-tender lump of size less than 5 cm in this study. Boral et al. [8] reported 51.64% of fibroadenomas involving the right breast, and the size of them ranged from 1 to 13 cm. Fibroadenoma with intracanalicular or pericanalicular growth pattern and without any other associated pathological findings is regarded as usual fibroadenoma, which accounted for 59.5% of the total cases in the present study, unlike the study by Yadav et al. [1], who reported 87.9% of usual fibroadenomas.

The unusual variants of fibroadenoma encountered in this study were complex fibroadenoma 65 (33.4%) and cellular fibroadenoma 4 (2.1%), while three (1.5%) cases of fibroadenoma were associated with tubular adenoma. Cellular fibroadenomas are characterized by increased stromal cellularity and have to be differentiated from benign phyllodes tumors, which show diffuse stromal cellularity that can be accentuated around epithelial clefts [9]. Complex fibroadenomas are characterized histologically by cysts (≥3 mm), sclerosing adenosis, papillary apocrine metaplasia, or epithelial calcifications [10]. The relative risk of development of invasive breast carcinoma was 2.17 times higher for patients with fibroadenoma than for matched controls, while the relative risk was 3.10 times higher in females with complex fibroadenoma [11].

Clinically, fibroadenomas more than 5 cm in size are regarded as giant fibroadenomas, which are commonly encountered in juvenile adolescent females [12]. Takei et al. [12] reported a statistically significant reduction in the size of fibroadenomas with the advancing age of females (p=0.0012). In the present study, fibroadenomas larger than 5 cm in size were often seen in females less than 20 years of age. However, the association between the age of the patients and tumor size was statistically insignificant (p=0.210).

Phyllodes tumors accounted for only 3.5% of the total fibroepithelial lesions encountered in this study, with the majority (five, 71.4%) of them occurring in females between age 21 and 40 years. These findings were almost similar to the studies of Yadav et al. [1] and Al-Atrooshi [2]. Unlike fibroadenomas, 85.7% of phyllodes tumors involved the left breast. In a study by Boral et al. [8], phyllodes tumors were often encountered in the right breast in contrast to the present study. In this study, 57.1% of the females with phyllodes tumors presented with a large breast lump of more than 10 cm in size, in concordance with the study of Al-Atrooshi [2], where the size of phyllodes tumors ranged from 2 cm to 20 cm, with most of them being larger than 10 cm in size.

Phyllodes tumors are graded into benign, borderline, and malignant as per the WHO criteria that include a degree of stromal cellularity, stromal overgrowth, cellular atypia, increased mitotic activity, and the nature of the tumor margins [13]. Benign phyllodes tumors exhibit circumscribed margins, mild stromal hypercellularity without stromal overgrowth, mild nuclear pleomorphism, and mitoses less than 5/10 high power fields. Malignant phyllodes tumors are characterized by infiltrative margins, stromal overgrowth associated with stromal hypercellularity, marked nuclear pleomorphism, and mitoses more than 10/10 high power fields. Borderline phyllodes tumors exhibit pushing margins and mitoses 5-9/10 high power fields with some but not all characteristics of malignant phyllodes tumors [13]. In the present study, benign and malignant phyllodes tumors accounted for 42.9% of cases each with one (14.2%) case of borderline phyllodes tumor. Yadav et al. [1] reported 61.3% of benign phyllodes tumors, 25.8% of borderline phyllodes tumors, and 12.9% of malignant phyllodes tumors. Similarly, Al-Atrooshi [2] encountered 66.7% of benign phyllodes tumors, 20% of borderline phyllodes tumors, and 13.3% of malignant phyllodes tumors. Thus in both these studies by Yadav et al. [1] and Al-Atrooshi [2], malignant phyllodes tumors were least common in contrast to the present study where borderline phyllodes tumors were least common.

In the present study, there was no significant association between the age of the patients and tumor size of phyllodes tumors (p=0.809) similar to the findings of Karim et al. [14]. It is postulated that changes in the hormonal milieu in older females may enhance the growth of phyllodes tumors [14]. Furthermore, in this study, the histological grade of phyllodes tumors did not show any significant association with the age group of patients (p=0.155) and tumor size (p=0.459). However, Yılmaz et al. [15] reported a statistically significant association between histological grade and age of patients (p=0.013) with the majority of borderline and malignant phyllodes tumors encountered in elderly females. There was also a significant association between histological grade and tumor size in the studies by Yılmaz et al. [15] and Slodkowska et al. [16] with most of the malignant phyllodes tumors being larger in diameter. This discordance could be attributed to the lower number of phyllodes tumors encountered in the current study.

Limitations

The major limitation of the study was the smaller number of phyllodes tumors encountered during the study period despite adequate sample size. This could be due to the overall rarity of these tumors in this population group and that the majority of patients encountered in this study were less than 40 years of age; phyllodes tumors are more common in elderly females. Further studies on fibroepithelial lesions including a greater number of phyllodes tumors will provide better insight into the clinicopathological characteristics of phyllodes tumors in comparison to fibroadenomas which are the most common fibroepithelial lesions of the breast worldwide.

## Conclusions

Fibroadenomas are the commonest fibroepithelial breast lesion followed by phyllodes tumors, exhibiting increased incidence among females in the reproductive age group, reflecting on the role of hormonal factors in the growth of these tumors, especially phyllodes tumors that often present as larger breast lumps. Histological grading of phyllodes tumors into benign, borderline, and malignant categories is based on WHO diagnostic criteria and helps differentiate benign phyllodes tumors from cellular fibroadenomas. Thus, fibroepithelial lesions are a broad group of commonly encountered biphasic tumors of the breast among females with overlapping histomorphological features. These tumors have to be diagnosed accurately based on detailed morphological analysis to provide appropriate treatment to the patients. The application of WHO morphological criteria will help in differentiating these lesions and arrive at a correct diagnosis based on thorough gross and microscopic examination.
